# siRNAs: Potential therapeutic agents against Hepatitis C Virus

**DOI:** 10.1186/1743-422X-8-276

**Published:** 2011-06-06

**Authors:** Usman A Ashfaq, Muhammad Z Yousaf, Maida Aslam, Rahat Ejaz, Shah Jahan, Obaid Ullah

**Affiliations:** 1 Division of Molecular Medicine, National Centre of Excellence in Molecular Biology, University of the Punjab, Lahore, Pakistan; 2 Institute of Biochemistry and Biotechnology, University of Veterinary and Animal Sciences, Lahore, Pakistan; 3 Applied and Functional Genomics Lab, Centre of Excellence in Molecular Biology University of the Punjab, Lahore, Pakistan

## Abstract

Hepatitis C virus is a major cause of chronic liver diseases which can lead to permanent liver damage, hepatocellular carcinoma and death. The presently available treatment with interferon plus ribavirin, has limited benefits due to adverse side effects such as anemia, depression and "flu-like" symptoms. Needless to mention, the effectiveness of interferon therapy is predominantly, if not exclusively, limited to virus type 3a and 3b whereas in Europe and North America the majority of viral type is 1a and 2a. Due to the limited efficiency of current therapy, RNA interference (RNAi) a novel regulatory and powerful silencing approach for molecular therapeutics through a sequence-specific RNA degradation process represents an alternative option. Several reports have indicated the efficiency and specificity of synthetic and vector based siRNAs inhibiting HCV replication. In the present review, we focused that combination of siRNAs against virus and host genes will be a better option to treat HCV

## Background

HCV infection is a serious global health problem that affects 180 million people worldwide and 10 million people in Pakistan [1]. Hepatitis C virus (HCV) causes acute and chronic hepatitis which can eventually lead to permanent liver damage, hepatocellular carcinoma and death [2]. Of those acutely infected with HCV, around 85% develop chronic infection. Approximately 70% of patients with chronic viremia develop chronic liver disease, 10-20% of which develop liver cirrhosis. It was estimated by the World Health Organization in 2004 that the annual deaths due to liver cancer caused by HCV and cirrhosis were 308,000 and 785,000, respectively [3].

HCV is a small enveloped virus with a positive sense, single-stranded RNA genome that encodes a large polyprotein of 3010 amino acids. The polyprotein is co- and posttranslationally processed by cellular and virally encoded proteases to produce four structural (Core, E1, E2 and P7) and six non-structural (NS2, NS3, NS4A, NS4B, NS5A, NS5B) proteins [4,5]. Among the structural protein, HCV envelop protein E1 and E2 are highly glycosylated and play an important part in cell entry. HCV NS3 serine protease and NS5b play an important role in replication. HCV NS3 serine protease, NS5B RNA-dependent RNA polymerase and HCV structural proteins are important targets for antiviral drug development.

There are six major and more than 80 subtypes of HCV. This classification is based on nucleotide variation among different HCV isolate. They occur in different proportion in different parts of the world. Genotype 1a and 1b are the most common genotypes in the United States and Europe [6,7]. The most prevalent HCV genotype in Pakistan is 3a followed by 3b and 1a [8].

Presently, there is no vaccine available for prevention of HCV infection due to high degree of strain variation. Current therapeutic options for hepatitis C are limited, especially for genotype 1. For genotypes 2 and 3, pegylated interferon in combination with ribavirin, can lead to a sustained virological response in up to 80% of patients [9]. However, the therapy is expensive and often associated with side effects that may lead to discontinuation of therapy [10]. Hemolytic anemia, cough, shortness of breath & treatogenicity are the most common adverse effect associated with ribavirin treatment, and muscle aches, fatigue & neuropsychiatric adverse effects of IFN-α lead to premature cessation of therapy in 10 to 20% of patients [11,12]. Moreover, cost of interferon for 6 month treatment ranging from 50,000 to 150,000 is beyond the financial range of most patients. Hence, there is need to develop anti HCV agents which are less toxic, more efficacious and cost-effective.

RNA interference (RNAi) is actually a sequence-specific RNA humiliation process in the cytoplasm of eukaryotic cells that is induced by double-stranded RNA (dsRNA). This RNA silencing mechanism, which was first described in *Caenorhabditis elegans *and *Drosophila melanogaster*, also possesses many similarities with post-transcriptional gene silencing in plants, and the process of quelling in *Neurospora crassa *[13,14]. RNAi and related RNA silencing mechanisms are supposed to act as a natural defense against incoming viruses and the expression of transposable elements [15]. Besides the antiviral function of RNAi, there is evidence that RNAi plays an important role in regulating cellular genes expression. These features have characterized RNAi both as an ancient and fundamentally important mechanism in eukaryotic cell biology [16]. Sequence-specific siRNA targeting of key human genes are ideal for studying protein function in cells. To recognize genes involved in a given cellular process, cell can be transfected with different siRNA and assayed for different response profiles.

### Mechanism of Silencing

**Figure 1:**
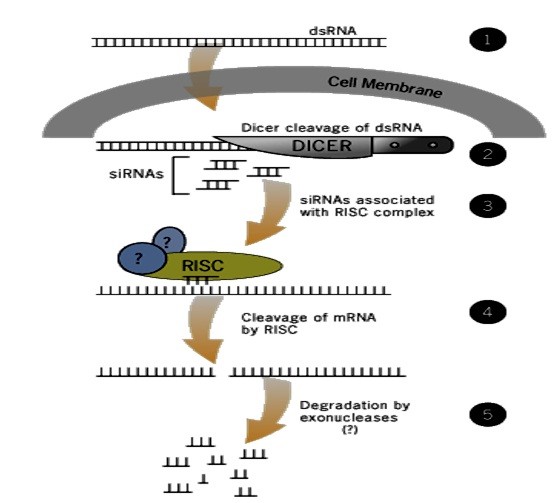
**How RNAi suppresses genes**: Short siRNA pieces unwind into single strand RNAs, which then combine with proteins to form RISC. The RISC then binds with a native mRNA molecule that complements the short siRNA sequence. If the pairing of native mRNA and siRNA piece is essentially perfect, the native mRNA is cut into useless RNA fragments that can't be translated. However, if the nucleotide base pairing is less than perfect, then the RISC complex binds to the mRNA and prevents the ribosome movement along the mRNA, also halting the translation, net resulting in no protein tranlation. (http://www.ambion.com/techlib/tn/101/7.html, Last Accessed: 9th April, 2010)

RNAi is exploits the conserved, powerful gene regulation pathway, by which dsRNA molecules are used to trigger the catalytic degradation of the targeted gene's mRNA, thereby effectively silencing its expression. The underlying RNAi process can be briefly summarized as follows: (Figure 1)

1. Firstly a "trigger"dsRNA is introduced into the cell's cytoplasm.

2. Second, is the generation of siRNA pool by the Dicer enzyme (and associated co-factors), which processes the trigger dsRNA and forms a pool of small interfering RNAs (siRNAs); these are ~21 base pairs in length, double stranded and include 2 nucleotide overhangs at both 3' ends.

3. Thridly, the processed siRNAs are then delivered to an Argonaut-containing RNA-Induced Silencing Complex (RISC), which unwinds the two siRNA strands, retaining one strand to act as a RISC-targeting co-factor.

4. Fourthly, the siRNA-associated RISC bind to the target mRNA, through the bound siRNA which confers sequence-based specificity to the associated RISC complex, allowing recognition and base-pairing with the complementary target mRNA.

5. And lastely, RISC complex contains an endonuclease activity, which is attributed to the Argonaut subunit, causes a single-site cleavage of the target mRNA approximately in the middle of the siRNA binding region. The resulting fragments of target mRNA is thus destabilized and subsequently gets fully degraded through natural endogenous mechanism[17].

### RNAi is a useful tactic in future Therapeutics

As whole human genome have been sequenced there is need to develop significant tools understanding the functions of specific genes, for this reason the need for a tool like siRNA is important in the therapeutic applications for a number of diseases [18]. Transcription, post-transcriptional and post-translational interventions are the three main time points at which a disease can be stopped. Before the discovery of antisense RNA most of the drug targets have been proteins and post-translation intervention [19]. siRNA is away to control the development and progression of diseases earlier on in the process. The high specificity of siRNA to target RNA makes it an important mechanism or tool to find out the function of the gene [20]. Use of RNAi along with "plasmid transfection technology with inducible vectors" like RNA pol III plasmid systems will make it possible to silence the effects of genes temporarily [19]. The possibility to accomplish RNAi based gene regulation in transgenic organisms has stimulated many explorations if this would be a useful option for medical therapy [21]. Results reported from a number of model organisms are quite promising [22-25] and even in recent clinical trials, but it is quite early to foretell the outcome of these challenging efforts.

Longer dsRNAs (50 bp) have a more broad effect in mammalian somatic cells, resulting in general arrest of protein synthesis through interferon response and also protein kinase activation. In contrast, shorter siRNAs of 21-23 nt have a more specific effect, inducing up to 90% suppression of specific mRNAs both *in vitro *and *in vivo *[26,27]. Due to its high suppression efficiency and sequence specificity up to a single nucleotide resolution [28] has encouraged the development of RNAi-based therapeutic models for possible use in viral infections i.e. HIV-1 [29], HBV [30], HCV [27], respiratory viruses [31] and cancer i.e. K-ras [32], PI 3-kinase [33]. The utilization of short double-strand RNA has completely eliminated the interferon response and non-specific mRNA degradation resulting due to long double-strand anti-sense RNA (>500 nucleotides) in the cytoplasm. Now, short nucleotides are being used for RNAi and have been adapted for high-throughput use for the transient knockdown of gene expression in cell lines and animals.

### RNAi as a therapeutic agent against HCV

Currently, there is no vaccine available for prevention of HCV infection due to high degree of strain variation. The current treatment of care, Pegylated interferon α in combination with ribavirin is costly, has significant side effects and fails to cure about half of all infections. HCV RNA is an attractive target for RNAi, as the single positive-stranded viral transcript functions both as genomic RNA and a replication template, and also because of its localization in the infected liver, an organ that can be readily targeted by nucleic acid molecules and viral vectors. As Dicer and the RISC act in the cytoplasm so the cytoplasmic location of RNAi machinery makes it technically easier than other methods that attempt to silence genes at the nuclear level. The gene silencing effects of synthesized siRNA is transient and is typically effective for approximately 3-7 days before they naturally disappear [34]. So there are little chances of side effects as with other therapeutic methods such as IFNα and ribavirin.

Several reports demonstrated potent RNAi activity against HCV in sub-genomic replicon and fully infectious HCV particles [12]. Synthetic or vector based siRNAs targeted against 5' untranslated region (UTR), HCV core, NS3, NS4B and NS5B were effective in reducing viral replication and infection [4,27,35-37]. The IRES containing conserved 5' UTR that is required for translation, has also been targeted by siRNA. Synthetic siRNAs and vector-derived shRNA against the 5' UTR resulted in 80% inhibition of HCV at a concentration of 2.5 nM [38]. siRNA targeted against 5' UTR of HCV sub-genomic replicon with the luciferase gene can also reduce the level of luciferase activity in a dose-dependent manner up to 85% to 90% [39]. In another report shRNA targeted to the 5' UTR inhibited virus replication and infectivity titers against HCV genotypes 1a and 2a [40]. Moreover, 5' UTR consensus siRNAs were designed and showed suppression of HCV genotype-4 replication *in vitro *in HCV serum infected Huh-7 cells [41]. shRNAs suppressing the HCV internal ribosomal entry site (IRES) inhibiting different HCV genotypes grown in cell culture and replicon replication has also been reported [42]. While the majority of HCV-siRNAs are complementary to the (+) strand, a reduction in both strands of the viral dsRNA replication intermediate has been observed [43]. It is plausible that targeting the (+) strand template indirectly leads to a decrease in synthesis of (-) strands. Two different groups used siRNA against Core gene of HCV 1a and 1b genotype and observed 60% and 80% reduction in mRNA and protein expression respectively [44,45]. A study demonstrated that siRNA targeted against E2, NS3 and NS5B regions effectively inhibit core gene expression [44] and Kim et al., 2006, has designed siRNA against HCV 1b and 1a genome to explore the silencing of structural genes and showed significantly less expression in a dose-dependent manner. A fragment of the HCV NS5B RNA polymerase gene, which was transiently co-transfected with siRNA into mouse liver by hydrodynamic injection, was reported to be cleaved after treatment with siRNA [27]. A number of laboratories have shown that siRNA targeted against protein coding regions of HCV can inhibit virus replication and expression [4]. It has been reported that simultaneous transfection with hairpin ribozymes directed against the 3'UTR HCV region and SiRNAs targeting the IRES domain triggers the efficient inhibition (up to 90%) of HCV replication in subgenomic replicon systems [46]. Although HCV mouse models are very limited, some groups have reported RNAi of HCV transgene expression in mice. One *in vivo *mouse study reported shRNAs specific to the HCV 5'UTR were effective at diminishing HCV internal ribosome entry site (IRES)-driven luciferase expression [47]. Another study using a similar method in mice reported silencing of a NS5B-luciferase transgene by NS5B-specific siRNAs [27].

Because dsRNAs can activate the interferon (IFN) pathway, it was necessary to address whether HCV-specific siRNAs could trigger IFN production. Kapadia *et al. *demonstrated that inhibition of viral replication by HCV RNAi *in vitro *was not associated with an up-regulation of IFN-stimulated genes [35]. In fact, HCV siRNAs were better at reducing HCV RNA levels than high doses of IFN-α [35]. An analysis of combined RNAi and IFN treatment of HCV replicons in cell culture using lentivirus-delivered shRNAs has been performed and results indicated that IFN-α enhance gene silencing, and inhibition of HCV replication [48]. This underscores the possibility of combination therapies of siRNAs and IFN against HCV. According to the reports, almost all regions of HCV show potential for siRNA target with relative efficiencies of individual siRNA sequences.

### Combination therapy of siRNA against host cellular genes involved in HCV infection

Host genes modulate viral infection and are an important target for antiviral therapy against HCV. Besides targeting HCV replication different studies demonstrated the feasibility of host cellular factor involved in infection, as they are not prone to mutations, as potential targets for siRNA therapy. Many groups have identified hundreds of host factors utilized by HCV in cell entry, replication and translation [49-53]. Host proteins that interact with the structural genes include CD81, SRBI, Claudin I and occludin [54-56]. CD81 is a tetraspanin that promotes HCV entry via its interaction with HCV E2 and Silencing CD81 with CD81 specific siRNA has blocked HCV entry in cells [57]. Down-regulation of Scavenger receptor class B type I (SR-BI) expression by SR-BI-specific siRNA markedly reduced the susceptibility of human hepatoma cells to HCV infection [58].

Randall and colleagues in 2003 determined whether HCV expression is interfered with siRNA against cellular (Lamin A/C) and viral RNA into Huh-7.5 cells containing replicating HCV. Both viral and cellular RNAs were efficiently silenced up to 80%. The efficiency of silencing lamin A/C expression was similar either in presence or absence of replicating HCV RNAs. Hepatitis B and C viruses are not cytopathic, but triggers hepatitis when activate immune cells expressing FasL, infiltrating the liver, where infection up regulates the death receptor Fas on hepatocytes, making them major targets for immune cells and might provide an effective immune modulating therapy to avoid chronic liver cell damage [59]. The administration of siRNA and shRNA to target cellular factor caspase 8, and NS5b has resulted in the destruction of cognate mRNA and protection of mice from liver failure [60].

Identification of cellular proteins with enzymatic functions is ideal for development of novel, small molecule inhibitors and/or therapeutic siRNAs. Two such candidates are ubiquitin specific peptidase 18 (USP18) and phosphatidylinositol 4-kinase III alpha (PI4K-IIIα). siRNAs targeting USP18 have been shown to potentiate the ability of IFN-α to inhibit HCV replication and virus production [Randall, 2006]. PI4K-IIIα siRNAs dramatically reduce HCV replication suggesting this is a critical viral replication cofactor [51-53]. Hsp90 is an important host derived factor that regulates HCV replication, siRNA against it inhibits the HCV replication in replicon cells and humanized mice liver [61].

HCV and triglyceride rich very low-density lipoproteins (VLDLs) both are secreted uniquely by hepatocytes in the form of membrane vesicles, highly enriched in proteins required for VLDL assembly, including apolipoprotein B (apoB), apoE, and microsomal triglyceride transfer protein, and circulate in blood in a complex. HCV production was reduced in hepatoma using an inhibitor of microsomal triglyceride transfer protein and siRNA directed against apoB indicating the possible explanation for the restriction of HCV production to the liver and suggest new cellular targets for treatment of HCV infection [62]. Several siRNAs targeted against human VAMP-associated protein (hVAP-A), La antigen and polypyrimidine tract binding protein (PTB) markedly decreased the expression levels of corresponding cellular genes that inhibited HCV replication in Huh-7 cells showing no impact upon cell viability [63]. Combinations of siRNAs directed against cellular HCV cofactors and HCV itself have revealed additive HCV RNA inhibition effects. Targeting multiple sites of the HCV genome and host factors involved in HCV replication are a realistic and valid approach aimed at preventing the virus from developing resistance.

## Conclusion

HCV causes acute and chronic hepatitis which eventually lead to permanent liver damage hepatocellular carcinoma and death. Current therapy for HCV infection is mainly the combination use of interferon and ribavirin, but only about half of the treated patients obtain a sustained antiviral response. Hence, the development of new therapies for HCV infection is urgent. Inhibitory properties of siRNAs on several components of HCV life cycle have provided a new approach for antiviral therapy. In the present review, we focused that combination of siRNA against virus and host genes will be better option to treat HCV

## Abbreviations

**HCV**: Hepatitis C virus; **siRNA**: Small interference RNA.

## Competing interests

The authors declare that they have no competing interests.

## Authors' contributions

UAA design the study and write up the manuscript. MZY, MA, RE, SJ and OU helped me in manuscript write up. All the authors read and approved the final manuscript.

## Authors' information

Usman A Ashfaq (PhD Molecular Biology), Muhammad Z Yousaf (PhD Molecular Biology), Maida Aslam (Mphil Chemistry), Rahat Ejaz (MSc microbiology) and Shah Jahan (PhD Molecular Biology)
